# Practical Utility of Liquid Biopsies for Evaluating Genomic Alterations in Castration-Resistant Prostate Cancer

**DOI:** 10.3390/cancers15102847

**Published:** 2023-05-20

**Authors:** Seung-Hwan Jeong, Dongsoo Kyung, Hyeong Dong Yuk, Chang Wook Jeong, Wookjae Lee, Jung-Ki Yoon, Hwang-Phill Kim, Duhee Bang, Tae-You Kim, Yoojoo Lim, Cheol Kwak

**Affiliations:** 1 Department of Urology, Seoul National University Hospital, Seoul 03080, Republic of Korea; 2 IMBdx Inc., Seoul 08506, Republic of Korea; 3 Department of Chemistry, Yonsei University, Seoul 03722, Republic of Korea; 4 Department of Internal Medicine, Seoul National University Hospital, Seoul 03080, Republic of Korea; 5 Cancer Research Institute, Seoul National University Hospital, Seoul 03080, Republic of Korea

**Keywords:** castration-resistant prostate cancer, liquid biopsy, cfDNA, genomic profiling, homologous recombination repair genes

## Abstract

**Simple Summary:**

The detection of specific genomic alterations is growing in importance for therapeutic decision-making in advanced prostate cancer. Traditional methods using tumor tissue samples can be challenging for prostate cancer, as the disease is often characterized by an extended disease history and a propensity for metastasis to the bone. Given the circumstances, liquid biopsy can be an attractive alternative. In this study, we evaluated the clinical usefulness of a liquid biopsy using blood samples instead of tumor tissues. The results showed that the liquid biopsy was able to evaluate cancer-related genomic changes in most patients, and it successfully detected clinically important mutations, exhibiting the high sensitivity of liquid biopsy compared to the tissue sequencing results. The liquid biopsy also enabled a genomic evaluation in cases where it would have been not possible using only archived tumor tissue samples. This study suggests that a liquid biopsy can be a good option for checking gene changes in advanced prostate cancer patients.

**Abstract:**

Traditional tissue-based assessments of genomic alterations in castration-resistant prostate cancer (CRPC) can be challenging. To evaluate the real-world clinical utility of liquid biopsies for the evaluation of genomic alterations in CRPC, we preemptively collected available plasma samples and archival tissue samples from patients that were being treated for clinically confirmed CRPC. The cell-free DNA (cfDNA) and tumor tissue DNA were analyzed using the AlphaLiquid^®^100-HRR panel. Plasma samples from a total of 87 patients were included in this study. Somatic mutations from cfDNA were detected in 78 (89.7%) patients, regardless of the presence of overt metastasis or concomitant treatment given at the time of plasma sample collection. Twenty-three patients were found to have known deleterious somatic or germline mutations in HRR genes from their cfDNA. Archival tissue samples from 33 (37.9%) patients were available for comparative analysis. Tissue sequencing was able to yield an NGS result in only 51.5% of the tissue samples. The general sensitivity of cfDNA for detecting somatic mutations in tissues was 71.8%, but important somatic/germline mutations in HRR genes were found to have a higher concordance (100%). Liquid biopsies can be a reasonable substitute for tissue biopsies in CRPC patients when evaluating genomic alterations.

## 1. Introduction

Prostate cancer is the second most common cancer in men and one of the leading causes of cancer-related mortality worldwide [1,2]. The main treatment strategy for advanced prostate cancer involves various ways in which to block androgen signals; however, in most cases, the disease eventually progresses to castration-resistant prostate cancer (CRPC) which is refractory to ADT treatment and may result in dismal consequences [3,4].

About 20 to 30% of metastatic prostate cancers harbor deficits with regard to homologous recombination repair (HRR); this commonly occurs as a result of mutations in relevant genes such as *BRCA2*, *BRCA1*, and *ATM*. Prostate cancers that harbor such mutations are known to show more aggressive behaviors [5]. Cancer cells with a loss of function in genes associated with HRR cause repair breaks in double-stranded DNA through relatively error-prone methods such as nonhomologous end joining. Thus, prostate cancers harboring mutations in HRR genes can be targeted with poly (adenosine diphosphate-ribose) polymerase (PARP) inhibitors via synthetic lethality [6,7]. The PARP inhibitors, olaparib and rucaparib, have been approved for the treatment of metastatic CRPC with HRR mutations [8,9]. Testing for mutations in HRR genes is important for selecting patients eligible for PARP inhibitor therapies, but practically, it is challenging in prostate cancer as primary tumor biopsy tissues are usually scant in volume, and metastatic lesions predominantly exist in bones [10,11].

Liquid biopsies using cell-free DNA (cfDNA) are emerging as a practical alternative to traditional tissue-based biopsies. Compared with a tissue biopsy, a liquid biopsy has several advantages related to the fact that it is easier and safer to obtain samples. It enables genomic evaluation in cases that have difficulties with regard to tumor tissue acquisition. Furthermore, as liquid biopsies enable repeated testing, the most relevant mutational status reflecting temporal evolution after treatment could be assessed even in cases that only have initial tissue biopsy results taken from a distant time-point [12,13].

This study was planned in order to show the real-world feasibility of genomic analysis using cfDNA in CRPC patients. This study aimed to evaluate the clinical applicability of liquid biopsies when assessing genomic variations in CRPC patients, including clinically important somatic and germline variants in HRR genes.

## 2. Materials and Methods

### 2.1. Study Participants

CRPC patients undergoing any type of systemic therapy at the Department of Urology at Seoul National University Hospital (SNUH) were preemptively enrolled for this study. Patients were allowed to participate in the study regardless of the status of their response to their current therapies. Participating patients provided 20mL of whole blood for cfDNA analysis. Tumor tissue samples were also collected from patients with archival tumor tissue samples that were available for research. For patients who did not have archival tissue samples available, the availability of results from the FIRST Cancer Panel (v.3., 183 genes), which is an NGS-based targeted panel sequencing platform that is used as part of the standard of care in SNUH, was checked, and the results were collected if permitted by the patients for research use.

### 2.2. Blood and Tissue Sample Processing

Each blood sample was collected using dedicated cfDNA bottles and each sample was centrifuged using a Ficoll solution at 1500× *g* for 15 min. Plasma was separated via centrifugation at 16,000× *g* for 10 min to remove cell debris. cfDNA was isolated from 2 to 4 mL plasma, in accordance with the manufacturer’s instructions, using a Maxwell^®^ RSC cfDNA Plasma Kit (Promega, Madison, WI, USA), and it was quantified using a 4200 TapeStation (Agilent Technologies, Santa Clara, CA, USA). Peripheral blood mononuclear cells (PBMC) were separated by following this protocol. Genomic DNA was isolated from PBMC using a Maxwell^®^ RSC Blood DNA Kit (Promega, USA). All experiments were processed in accordance with guidelines outlining the pre-analytical conditions for analyzing cfDNA [14,15,16]. 

Genomic DNA was isolated from tumor tissue samples using a Maxwell^®^ RSC FFPE DNA Kit (Promega, USA) for FFPE samples, and a Maxwell^®^ RSC Tissue DNA Kit (Promega, USA) for fresh-frozen tissues.

### 2.3. Targeted Panel Sequencing

The DNA NGS library was constructed using the IMBdx NGS DNA Library Prep Kit. Solution-based target enrichment was performed at IMBdx, Inc. (Seoul, Republic of Korea), using the AlphaLiquid^®^ 100 -HRR target capture panel. The targeted gene panel consists of 118 cancer-related genes, including 14 HRR-related genes, and it was designed to cover the entire exon of the genes (Supplementary Table S1). Captured DNA libraries were sequenced using the Illumina Novaseq 6000 platform (Illumina, San Diego, CA, USA) in a 2 × 150 bp paired-end mode. All sequencing reads from the samples were generated in a bcl format, and they were de-multiplexed into fastq files using the Burrows–Wheeler Aligner (BWA, version 0.7.17-r1188) [17]. The fastq files were trimmed for adaptor sequences and then aligned with the human reference genome (hg38) using BWA (version 0.7.10) “mem” algorithm. Reads mapped onto the AlphaLiquid^®^ 100 -HRR target regions were extracted. The median read counts on all 118 CDS (321,358 bps) for each sample were used to estimate the sequencing depth. Initial calls were made using fragment counts, which were single-strand consensus sequences (SSCS) and duplex consensus sequences (DCS) using fgbio tools (http://fulcrumgenomics.github.io/fgbio accessed on 3 February 2022). The molecular depth (X) was estimated by adding the SSCS and DCS counts for the CDS region. Calls were scored using a machine-learning model, distinguishing true variants from false variants, then, they were annotated for functional effect prediction. Sequencing procedures for tissue samples, either FFPE or fresh frozen, were processed using the same methods as blood samples.

### 2.4. Identifying cfDNA Genomic Alterations

After primary variant calling, we removed germline mutations and the variants that were considered to be the result of clonal hematopoiesis of indeterminate potential by comparing the PBMC results. Deleterious germline mutations occurring in the *BRCA1* or *BRCA2* genes were exceptions, and were not removed. We applied a cutoff for cfDNA mutations of VAF ≥ 0.1% and altered the DCS count ≥ 4. We also applied a cutoff for tissue mutations of VAF ≥ 10% to filter out noises, contaminations, or sequencing errors. For the determination of copy number alterations, a pre-built sequencing depth profile of 50 healthy subjects for the exonic regions targeted by the panel was used as a reference. The log2 ratios were calculated using the CNVkit [18]. We defined copy number (CN) gains as CN ≥ 4, along with statistical criteria that had *p*-values less than 0.01. A loss of heterozygosity (LOH) was defined by copy numbers that were less than 1.8, with a heterozygotic B-allele frequency of the segment of each HRR gene. For functional classification, the mutations that were determined as being “pathogenic” or “likely-pathogenic” on ClinVar Significance were defined as deleterious. Other mutations without known functional classifications, including those annotated as “Conflicting interpretations of pathogenicity” or “Uncertain significance”, were defined as being variants of unknown significance (VUS).

### 2.5. Prediction of HRR Deleterious Mutations

For VUS occurring in HRR genes, pathogenicity was predicted by using three independent algorithms, EVE [19], MetaRNN [20], and BayesDel [21]. These algorithms can predict the clinical significance of missense or small indel variants. Substitutions of DNA and amino acids, along with their positions, were imported for analysis using the three algorithms, and scores were given to each result: “1”, if predicted deleterious, “−1”, if predicted tolerated, and “0”, if no functional prediction was made. The final prediction of pathogenicity for each VUS of interest was ascertained using the sum of the scores from the three algorithms.

### 2.6. Statistical Analysis

To analyze the association between cfDNA results and various clinical variables, the Chi-squared test was used for the categorical variables, and the Student’s *t*-test was used for the continuous variables between the two groups. A *p*-value of less than 0.05 was considered statistically significant. The statistical analysis was performed using R (version 4.2.1) software.

## 3. Results

### 3.1. Patient Characteristics

A total of 87 patients with CRPC were included in this study. Patient characteristics are summarized in Table 1. The median age of the patients was 71 (range 53–89). Most of the included cases were high-grade prostate cancers of ISUP grade group 4 (27.5%) or 5 (56.3%), and the median level of the prostate-specific antigen (PSA) at the time of blood sample collection was 8 ng/mL (range 0–2277). Seventy-eight (89.6%) of the included cases were metastatic CRPC (mCRPC), although no radiological metastatic lesions were confirmed in 9 (10.2%) cases. Among the 79 mCRPC cases, the most frequently involved organ of metastasis was bone (79.3%), followed by distant lymph nodes (18.4%), lung (11.5%), and liver (5.7%). Forty-nine (71.0%) of the 69 patients with bone metastasis had bone as their only site of metastasis. The blood samples were collected at the time of progression, due to their previous line of treatments, in 40 (47.6%) of the 87 patients, whereas the samples were collected during the course of hormonal therapy in 37 patients (42.5%), and at the time of cytotoxic chemotherapy in 7 (8.0%) patients.

### 3.2. Detection of Somatic Mutations from cfDNA

The 87 plasma samples were sequenced at a median of 55G base pairs per sample and a median depth of 58,062× (95% CI 55,584–60,539). The on-target ratio was 60.5%. Somatic mutations were detected in the plasma samples of 79 (90.8%) of the patients, with a median of two mutations per patient (range 1–37). The most frequently found gene with somatic variants in all patients was AR (altered in 30% of patients), followed by TP53 (24%), CDK12 (17%), ATM (15%), BRCA2 (13%), and ROS1 (13%) (Supplementary Figure S1). Patients with a plasma PSA level higher than the median (≥8 ng/mL) at the time of cfDNA evaluation were more likely to have somatic mutations detected in their plasma samples (any somatic mutations detected in 79.5% (31/39) in PSA < 8 ng/mL vs. 97.7% (42/43) in PSA ≥ 8 ng/mL, *p* = 0.009). However, the rate of detection was not significantly affected by the presence of overt metastasis, with somatic ctDNA mutations detected in 77.8% (7/9) of the non-metastatic CRPC (nmCRPC) cases compared with 91.0% (7/71) of the mCRPC cases (*p* = 0.217). Among the mCRPC cases with bone as the only organ of metastasis, somatic mutations were detected in 93.9% (46/49) of the cases. Additionally, the rate of detection of somatic mutations in cfDNA was not affected by the type of treatment being given at the time of blood sample collection. cfDNA somatic mutations were detected in all 7 patients who were being treated with, and who were responding to, cytotoxic chemotherapy at the time of blood collection.

### 3.3. Variants in Homologous Recombination Repair Genes Detected by cfDNA

**Figure 1:**
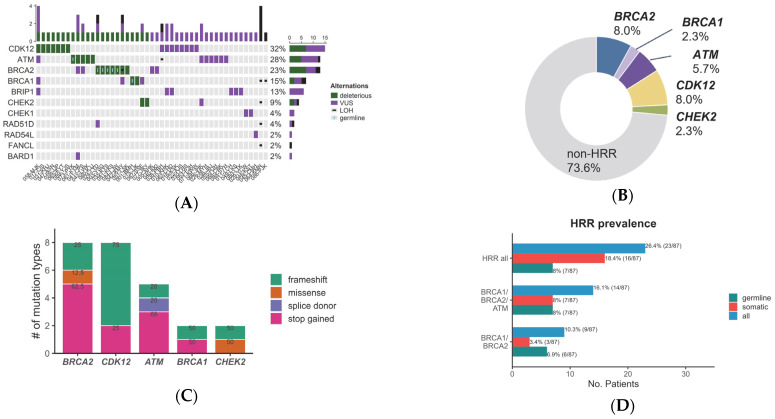
Genetic alteration of 14 HRR genes in cfDNA of CRPC patients. (**A**) The landscape of HRR mutations. (**B**) Prevalence of the deleterious mutations. (**C**) Fractions of types of deleterious mutations. (**D**) Frequency of germline and somatic mutations in HRR genes.

Focusing on the HRR genes which are clinically important, any mutations in HRR genes were detected in 47 (54%) of the patients for a total of 71 somatic and 7 germline mutations. The most frequently altered HRR gene was *CDK12* (17%), followed by *ATM* (15%), *BRCA2* (13%), *BRCA1* (8%), and *BRIP1* (7%) (Figure 1A). Of the 47 cases with HRR gene mutations, 23 (48.9%) patients were found to have deleterious mutations whereas 24 (51.1%) patients had VUS mutations only.

Counting only the deleterious mutations, the HRR gene that was found to have the most alterations was *CDK12* (10.3%), followed by *BRCA2* (9.2%), *ATM* (5.7%), *BRCA1* (2.3%), and *CHEK2* (2.3%) (Figure 1B). The mutation types of deleterious mutations in HRR genes were mostly frameshift or stop-gained mutations (Figure 1C). Germline mutations accounted for 30.4% of all deleterious mutations and were found in *BRCA2* (5 patients), *BRCA1* (1 patient), and *ATM* (1 patient) genes (Figure 1D).

**Figure 2:**
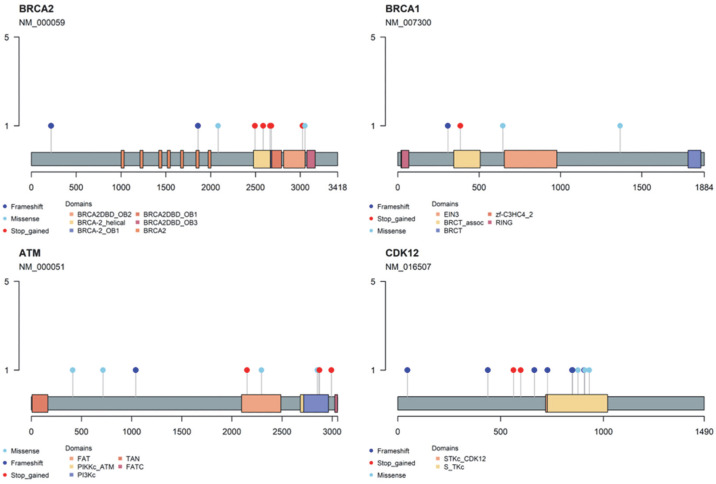
Location on major HRR proteins according to mutation type.

As a significant proportion of the alterations in HRR genes were VUS, we have predicted the pathogenicity of these alterations using three independent pathogenicity prediction algorithms. Among the 44 VUS evaluated, 16 (36.4%) alterations were consistently predicted to be deleterious by two or more algorithms (Supplementary Table S2). The mutations that were predicted to be pathogenic were posed at inter-domain and linker domains in *BRCA2*, *BRCA1*, *ATM*, and *CDK12* genes (Figure 2). Using these algorithms, 11 patients (12.6%) out of 24 patients with VUS only were additionally classified as having suspected deleterious mutations in HRR genes.

### 3.4. Comparison of cfDNA Analysis Results with Tissue Sample Results

Targeted sequencing was performed on 29 available tissue samples to evaluate the concordance of mutations detected between tissue DNA and plasma cfDNA. In four patients, results from the FIRST Cancer panel were adopted for the comparative analysis. The tissue samples were from primary prostate cancer in 31 patients, whereas two were from metastatic bone lesions. The time gap from tissue collection to blood collection ranged from 0–16 years, with a median of 2 years. Thirty-two somatic mutations were detected from tissue samples in 17 (51.5%) of the 33 patients. The samples that were archived for more than 3 years were more likely to fail to yield an NGS result (no mutations found in 10/14 (71.4%) of samples ≥ 3 years vs. 6/19 (31.6%) of samples < 3 years, *p* = 0.024).

Compared with the results from cfDNA, 23 mutations were identified in both plasma and tissue (sensitivity 71.8%). Nine mutations were only identified in tissue, and 53 mutations were only identified in plasma. Of the 32 tissue mutations, 15 were deleterious mutations, and 13 of the 15 deleterious mutations were also confirmed as being cfDNA mutations (sensitivity 86.7%). The two pathogenic mutations identified in tissue only were *KRAS* G12R in IMB001 and *TP53* R248W in IMB013. Moreover, 10 pathogenic mutations were found in the plasma only.

Twelve mutations in HHR genes were found in the 33 tissue samples, including 6 deleterious mutations (2 germlines, 4 somatic). The 6 deleterious mutations in the tissue samples were all found in cfDNA (sensitivity 100%). *ATM* L1042* was identified in the plasma of IMB043, but it was not confirmed as existing in tissue. There were no additional suspected deleterious mutations found in the tissue samples by algorithms, although there were two suspected deleterious mutations in the cfDNA of the 33 matching patients.

## 4. Discussion

In this study, we report the practical feasibility of using cfDNA for the analysis of genomic alterations in CRPC patients. The liquid biopsy was able to detect somatic mutations in 90.8% of all included patients, detecting mutations even in patients without overt metastasis or who were responding to other therapies, including chemotherapies. The liquid biopsy also detected deleterious somatic or germline alterations in the HRR genes of 26.4% of patients. An additional 12.6% of patients were suggested as carrying potentially sensitive mutations to PARP inhibitors by applying pathogenicity prediction algorithms, which could be the subject of further clinical investigation. On the other hand, archival tissue was available in fewer than 40% of the included patients and successful NGS analysis was possible in even fewer patients. The clinically important mutations revealed from tissue results were all covered by the liquid biopsy.

Assessments of genomic alteration status has become a practically important step for decision-making in CRPC treatment, especially with regard to the approval of PARP inhibitors in prostate cancer harboring mutations in HRR genes [8,9]. However, genomic analysis using traditional tissue-based methods can be practically challenging, especially due to the biological characteristics of prostate cancer. Primary prostate cancer tissues obtained during the initial diagnosis are often scant in amount, and the primary tissues obtained during surgery in cases where resection can initially occur are usually dated back years ago, to when a patient required systemic therapy decisions. Biopsies of metastatic lesions are especially challenging as prostate cancer tends to metastasize predominantly to the bone [10,11,22]. Biopsy procedures of the bone are not only technically challenging, but they can also be distressing to patients. Moreover, bone biopsies often yield suboptimal samples for genomic evaluation, as they often produce an inadequate amount of viable tumor cells, and the unique tissue processing procedures may further decrease the analyzable amount of DNA [23,24]. Other studies have reported biopsy failure rates higher than other cancer types, including a 31% failure rate in the biomarker-selected phase 3 trial PROfound [9,25,26].

In our study cohort, we have collected cross-sectional blood samples from patients undergoing treatment for their CRPC in our clinic. We reviewed the status of their archival tissue sample availabilities, and only 37.9% of patients had any archival tissue available for genomic analysis unless a new biopsy was performed. In addition, 33 (61.1%) of the 54 patients without any archival tumor tissues had bone as their only site of metastasis. Using cfDNA analysis, we were able to report genomic analysis results in 92.0% of the included patients, including the detection of somatic mutations in 78 patients and relevant germline BRCA1 or BRCA2 mutations in an additional 2 patients. Considering our findings, it is important to note that the utilization of the liquid biopsy enabled the acquisition of results in patients for whom genomic testing would have been impossible if traditional tissue-based approaches were used. Given the established significance of PARP inhibitors as an effective targeted agent in select patients with prostate cancer, increasing the number of patients who can undergo genetic testing in this manner represents a substantial advancement in patient treatment. Moreover, the detectability of genomic alterations in the cfDNA was not limited to patients who were expected to have large viable tumor volumes, thus indicating the flexibility of the application of liquid biopsies in CRPC patients.

We previously detected and annotated clinically relevant mutations in the HRR genes of 29.5% of all patients, which is comparable to other studies reporting HRR mutations in 25–30% of CRPCs patients [27,28,29]. However, a significant number of VUS in HRR genes were also found, and it cannot be concluded that the carriers of VUS, especially in *BRCA1* and *BRCA2* genes, will not respond to PARP inhibitors. It is important to note that the variant classification of VUS does not necessarily indicate non-functionality, but rather, it signifies that their function is unknown, and that the functional classifications of these VUS variants have been changing over time [30,31]. In our study cohort, 44 VUS of HRR genes were detected from 23 patients, of which, 16 (36.4%) were classified and estimated as being possibly deleterious. Although the accumulation of more evidence is necessary to conclude that the mutations specified by the algorithm are clinically actionable, there is emerging evidence of PARP inhibitor responses in these unknown mutations [32]. Increasing the incidence of such mutations for further investigation may provide future patients with a chance to be treated with efficacy.

Our study has limitations in that the analysis was performed on a relatively small number of patients. A comparison of analysis results between blood and tissue samples was only available in smaller, heterogeneous sample sets. Moreover, the study was not designed to link the detection of cfDNA variants, or the quantity of the variants detected, with the clinical outcomes in these patients. Future studies incorporating a larger patient population to assess the clinical outcomes of diverse treatments, with a particular emphasis on PARP inhibitor therapy based on the outcomes of ctDNA assessments, are warranted to strengthen the clinical utility of the ctDNA assay. However, the study results indicate that there is a benefit to conducting liquid biopsies in CRPC patients, thus reflecting the real-world limitations associated with the difficulties in acquiring adequate tissue samples.

## 5. Conclusions

A liquid biopsy can be a practical alternative to tissue-based genomic analysis in CRPC patients. By facilitating genomic analysis for drug selection in a greater number of patients through liquid biopsies, it contributes to the pursuit of personalized medicine. Further investigations, including a larger number of patients with treatment response data, are necessary.

## Figures and Tables

**Table 1:** Patient Characteristics.

	Number of Patients (%, N = 87)
Age at initial diagnosis	
Years (median, range)	71 (53–89)
Time from the initial diagnosis to plasma sample collection	
Months (median, range)	37 (0–268)
PSA at the time of plasma sample collection	
ng/mL (median, range)	8 (0–2277)
Pathologic diagnosis	
Adenocarcinoma	86 (98.9%)
Others (Squamous cell carcinoma)	1 (1.1%)
ISUP GG	
1–3	17 (19.5%)
4	24 (27.6%)
5	46 (52.9%)
Number of metastatic organs	
0 (nmCRPC)	9 (10.3%)
1	57 (65.5%)
≥2	21 (24.1%)
Organ of Metastasis	
Bone ^1^	69 (79.3%)
Distant lymph nodes	16 (18.4%)
Lung	10 (11.5%)
Liver	5 (5.7%)
Others	6 (6.9%)
Systemic treatments given at the time of plasma sample collection	
Hormonal agents	37 (42.5%)
Cytotoxic chemotherapy	7 (8.0%)
Before the beginning of the next line of therapies	40 (47.6%)
Information not available	3 (3.4%)

Abbreviations: PSA, Prostate-specific Antigen; ISUP GG, International Society of Urological Pathology Gleason Group; nmCRPC, non-metastatic castration-resistant prostate cancer. ^1^ Of all patients, 49/69 (71.0%) had bone as their only organ of metastasis.

## Data Availability

The data that supports the findings of this study are included in the supplementary materials of this article. The raw sequencing data have been deposited with links to BioProject accession number PRJNA930970 in the NCBI BioProject database (https://www.ncbi.nlm.nih.gov/bioproject/ accessed on 3 February 2023).

## Supplementary Materials

The following supporting information can be downloaded at: https://www.mdpi.com/article/10.3390/cancers15102847/s1, Figure S1: Mutation landscape of cfDNA in 87 CRPC patients; Table S1: Genes of the AlphaLiquid^®^100; Table S2: Predicted pathogenicity scores of the suspected and other VUS HRR mutations.
