# Supplementary figures and images for: Practical Utility of Liquid Biopsies for Evaluating Genomic Alterations in Castration-Resistant Prostate Cancer
Source: Cancers (Basel). 2023 May 20;15(10):2847. doi: 10.3390/cancers15102847 (PMC10216019; doi:10.3390/cancers15102847)

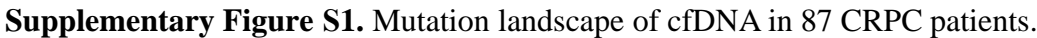

**Supplementary Figure S1.** Mutation landscape of cfDNA in 87 CRPC patients.

Supplement: Supplementary file 1 [file cancers-15-02847-s001.zip › Supplementary Figure S1.pdf]
